# Worldwide Incidence of Malaria in 2009: Estimates, Time Trends, and a Critique of Methods

**DOI:** 10.1371/journal.pmed.1001142

**Published:** 2011-12-20

**Authors:** Richard E. Cibulskis, Maru Aregawi, Ryan Williams, Mac Otten, Christopher Dye

**Affiliations:** 1 Global Malaria Programme, HIV/AIDS, Tuberculosis, Malaria & Neglected Tropical Diseases Cluster, World Health Organization, Geneva, Switzerland; 2 Office of Health Information, HIV/AIDS, Tuberculosis, Malaria & Neglected Tropical Diseases Cluster, World Health Organization, Geneva, Switzerland; Walter & Eliza Hall Institute, Australia

## Abstract

**Background:**

Measuring progress towards Millennium Development Goal 6, including estimates of, and time trends in, the number of malaria cases, has relied on risk maps constructed from surveys of parasite prevalence, and on routine case reports compiled by health ministries. Here we present a critique of both methods, illustrated with national incidence estimates for 2009.

**Methods and Findings:**

We compiled information on the number of cases reported by National Malaria Control Programs in 99 countries with ongoing malaria transmission. For 71 countries we estimated the total incidence of *Plasmodium falciparum* and *P. vivax* by adjusting the number of reported cases using data on reporting completeness, the proportion of suspects that are parasite-positive, the proportion of confirmed cases due to each *Plasmodium* species, and the extent to which patients use public sector health facilities. All four factors varied markedly among countries and regions. For 28 African countries with less reliable routine surveillance data, we estimated the number of cases from model-based methods that link measures of malaria transmission with case incidence. In 2009, 98% of cases were due to *P. falciparum* in Africa and 65% in other regions. There were an estimated 225 million malaria cases (5th–95th centiles, 146–316 million) worldwide, 176 (110–248) million in the African region, and 49 (36–68) million elsewhere. Our estimates are lower than other published figures, especially survey-based estimates for non-African countries.

**Conclusions:**

Estimates of malaria incidence derived from routine surveillance data were typically lower than those derived from surveys of parasite prevalence. Carefully interpreted surveillance data can be used to monitor malaria trends in response to control efforts, and to highlight areas where malaria programs and health information systems need to be strengthened. As malaria incidence declines around the world, evaluation of control efforts will increasingly rely on robust systems of routine surveillance.

*Please see later in the article for the Editors' Summary*

## Abstract 2

Richard Cibulskis and colleagues present estimates of the worldwide incidence of malaria in 2009, together with a critique of different estimation methods, including those based on risk maps constructed from surveys of parasite prevalence, and those based on routine case reports compiled by health ministries.

## Editors' Summary

**Background:**

Malaria is a life-threatening disease caused by the Plasmodium parasite, which is transmitted to people through the bites of infected mosquitoes. According to latest estimates from the World Health Organization (WHO), in 2009, there were 225 million cases of malaria and an estimated 781,000 deaths worldwide—most deaths occurring among children living in the WHO African Region (mainly sub-Saharan Africa). Knowing the burden of malaria in any country is an essential component of public health planning and accurately estimating the global burden is essential to monitor progress towards the United Nations' Millennium Development Goals.

Currently, there are generally two approaches used to estimate malaria incidence:

One method uses routine surveillance reports of malaria cases compiled by national health ministries, which are analyzed to take into account some deficincies in data collection, such as incomplete reporting by health facilities, the potential for overdiagnosis of malaria among patients with fever, and the use of private health facilities or none at all. The second method uses population-based surveys of Plasmodium prevalence and case incidence from selected locations in malaria endemic areas and then uses this information to generate risk maps and to estimate the case incidence of malaria per 1,000 population, for all of the world's malaria endemic regions. The Malaria Atlas Project—a database of malaria epidemiology based on medical intelligence and satellite-derived climate data—uses this second method.

**Why Was This Study Done?:**

In order for malaria epidemiology to be as accurate as possible, an evaluation of the strengths and weaknesses of both methods is necessary. In this study, the researchers analyzed the merits of the estimates calculated by using the different approaches, to highlight areas in which both methods need to be improved to provide better assessments of malaria control.

**What Did the Researchers Do and Find?:**

The researchers estimated the number of malaria cases in 2009, for each of the 99 countries with ongoing malaria transmission using a combination of the two methods. The researchers used the first method for 56 malaria endemic countries outside the WHO African Region, and for nine African countries which had the quality of data necessary to calculate estimates using the researchers statistical model—which the researchers devised to take the upper and lower limits of case detection into account. The researchers used the second method for 34 countries in the African Region to classify malaria risk into low-transmission and high-transmission categories, and then to derive incidence rates for populations from observational studies conducted in populations in which there were no malaria control activities. For both methods, the researchers conducted a statistical analysis to determine the range of uncertainty.

The researchers found that using a combination of methods there was a combined total of 225 million malaria cases, in the 99 countries malaria endemic countries—the majority of cases (78%) were in the WHO African region, followed by the Southeast Asian (15%) and Eastern Mediterranean regions. In Africa, there were 214 cases per 1,000 population, compared with 23 per 1,000 in the Eastern Mediterranean region, and 19 per 1,000 in the Southeast Asia region. Sixteen countries accounted for 80% of all estimated cases globally—all but two countries were in the African region. The researchers found that despite the differences between methods 1 and 2, the ratio of the upper and lower limit for country estimates was approximately the same.

**What Do These Findings Mean?:**

Using the combined methods, the incidence of malaria was estimated to be lower than previous estimates, particularly outside of Africa. Nevertheless the methods suggest that malaria surveillance systems currently miss the majority of cases, detecting less than 10% of those estimated to occur globally. Although the best assessment of malaria burden and trends should rely on a combination of surveillance and survey data, accurate surveillance is the ultimate goal for malaria control programs, especially as routine surveillance has advantages for estimating case incidence, spatially and through time. However, as the researchers have identified in this study, strengthening surveillance requires a critical evaluation of inherent errors and these errors must be adequately addressed in order to have confidence in estimates of malaria burden and trends, and therefore, the return on investments for malaria control programs.

**Additional Information:**

Please access these Web sites via the online version of this summary at http://dx.doi.org/10.1371/journal.pmed.1001142.

- This study is further discussed in a *PLoS Medicine* Perspective by Ivo Mueller and colleagues
- The WHO provides information on malaria and produces the World Malaria Report each year, summarizing global progress in malaria control
- More information is available on The Malaria Atlas Project

## Introduction

Knowing the number of malaria cases that occur annually in any country is an essential component of planning national health services and evaluating their effectiveness. Reliable data from each endemic country are needed to assess progress globally towards the United Nations Millennium Development Goals. At present there are broadly two approaches to estimating malaria incidence country by country. One method uses routine surveillance reports of malaria cases compiled by health ministries, adjusted to take into account incomplete case detection by health facilities, the potential for overdiagnosis of malaria among patients with fevers, and the way patients use public and private health services [1]. The second, cartographic method uses population-based surveys of parasite prevalence and case incidence from selected locations to generate, by extrapolation, risk maps (i.e., maps of case incidence per 1,000 population) across malaria endemic regions of the world. This second method is favoured by the Malaria Atlas Project (MAP) [2]–[8]. A major challenge for malaria epidemiologists is to evaluate the strengths and weaknesses of both methods in estimating malaria incidence and time trends, especially as malaria control programmes are intensified worldwide. Other related work has focused on the subset of cases that are relatively severe (e.g., severe malarial anaemia, cerebral malaria, neurological sequelae) [9] and on deaths due to malaria, and is not discussed further here.

The most recent presentation of estimates made primarily by cartography (from MAP) [4] gives point estimates of 271 million *P. falciparum* malaria cases in 47 countries on the African continent and 180 million *P. falciparum* cases in other countries during 2007. Those estimates were based on national case reports from seven countries, and on risk maps for 80 countries. Here we present another assessment of the worldwide distribution of malaria incidence, for 2009, using a combination of routinely collected case reports (for 65 countries, mainly outside Africa) and risk maps (for 34 countries with less reliable reporting from surveillance systems, all in Africa), and allowing for the rapid increases in coverage of insecticide-treated nets since 2005. Compared with MAP estimates for 2007 [4], our analysis yields lower estimates for most countries, and especially for several major endemic countries outside Africa. We discuss the validity of estimates obtained using the two different approaches, and highlight areas in which both methods need to be improved to provide better assessments with which to evaluate efforts to control malaria.

This study includes a critique of methods used to assess the scale of the malaria problem worldwide, illustrated with estimates derived by the two principal methods. Besides making some allowance for vector control, we do not attempt to explain the geographical and temporal distribution of malaria cases in terms of the characteristics of vectors, hosts, and environment; that would require additional data and further work.

## Methods

The estimation methods used in this study are described briefly below and fully in Text S1. Countries are allocated to the six regions defined by the World Health Organization (WHO).

### Estimating the Incidence of Malaria Cases

A case of malaria was defined as fever with *Plasmodium* infection (blood smear or rapid diagnostic test [RDT]), which identifies individuals who require antimalarial treatment. Of the 106 countries most affected by malaria, seven are in WHO's “prevention of reintroduction” phase during which there is no local transmission. In this study estimates of the number of malaria cases were made for each of the 99 countries with ongoing malaria transmission, by one of two methods.

#### Method 1: Estimates from routine case reports (surveillance)

Upper and lower limits for the estimated number of cases, *M*, arising in any given year in a country are calculated from:
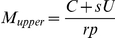


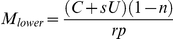
Where: *C* = reported number of confirmed malaria cases in a year; *U* = reported number of unconfirmed cases in a year: cases suspected of being malaria but not tested or confirmed, sometimes known as probable cases; *s* = the proportion of slides examined that is positive for malaria parasites (slide positivity rate) or the proportion of RDTs that gives a positive result; *r* = completeness of health-facility reports. This is the number of outpatient health-facility reports received divided by the number of facility reports expected. The expected number of reports is the number of health facilities multiplied by the number of reports expected to be submitted by each health facility in a year, which is 12 for a monthly reporting system; *p* = the proportion of the population with fever (or suspected malaria) that uses health facilities that are covered by the public health-facility reporting system. This was derived from household survey data describing whether or not children under 5 y, with fever in the previous 2 wk, sought treatment and where. The household survey used for most countries was a Demographic and Health Survey (DHS) or Multiple Indicator Cluster Survey (MICS); *n* = the proportion of fever cases (or suspected malaria) that do not seek treatment. This was derived from household survey data, as for *p*.

**Figure 1:**
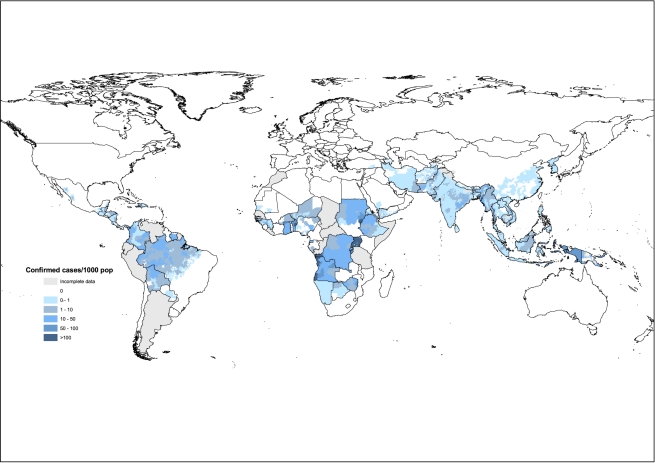
Distribution of confirmed malaria cases per 1,000 population, for the lowest administrative level possible in each country. The number of countries providing data at different administrative levels (from national level 0 down to subnational level 5) were: level 0, 13; level 1, 71; level 2, 19; level 3, 2; level 4, 0; level 5, 1. The total of 106 countries affected by malaria, includes the 99 with ongoing transmission, and seven in the WHO “prevention of reintroduction” phase. Where national data were incomplete, the whole country is marked as such on the map.

Values of *C*, *U*, *n*, *p*, *r*, *and s* are given for each country in Table 1 and Text S1. Figure 1 shows the distribution of confirmed cases worldwide, for the lowest administrative level possible in each country (typically, administrative level 1 in Africa, but down to administrative level 5 in Brazil). The difference between upper and lower limits of *M* reflects the extent to which malaria cases are treated in the health system, both formal and informal. The upper limit is an estimate of the number of malaria cases assuming the same slide positivity rate, *s*, among those who do and do not seek treatment. The lower limit estimates the number of malaria cases if only those fever cases that seek treatment have malaria (i.e., *s* = 0 for fever cases not seeking treatment). In practice the true value will probably lie between these points. It will lie close to the lower limit in areas where accessibility to services is good and all cases that need treatment actually seek it. It will lie closer to the upper limit in areas where accessibility of services is poor, and many malaria cases go untreated. In the absence of detailed information on the structure of health services in a country, we derived a single point estimate, *M*, from the arithmetic of average of *M*_lower_ and *M*_upper_. Method 1 was used for all 56 non-African malaria endemic countries, and for nine African countries for which the quality of data were considered adequate.

**Table 1:** Distributions assumed for parameters used in method 1.

Parameter	Assumed Distribution	Description				
	Parameter derived from reported data					
*r*	For each value of reporting completeness, *r* was assumed to be distributed as follows:	Reported Value	Distribution	Minimum	Most Likely	Maximum
		80%+	Triangular	80%	80%	100%
		50–80%	Uniform	50%	—	80%
		<50%	Triangular	0%	50%	50%
*s*		The uncertainty analysis aimed to reflect the variation of *s* within a country, so that when *s* was applied to cases that were not microscopically examined the slide positivity rate could take on a range of values that could reasonably be expected to occur across the country. Specifically, the national slide positivity rate, *s*, was assumed to be distributed normally with a mean c and standard deviation of 0.311*s* ^0.5547^. Values of *s* were then truncated so that values lie between 0 and 1. This relationship was obtained from a least squares regression of the mean value of *s* against the standard deviation of *s* for each country for which subnational values of *s* were available.				
*p* and *n*		*p* and *n* were assumed to be distributed normally with mean and standard deviation derived directly from analysis of household surveys, taking into account the specified sampling design.				
Parameter	If parameter imputed					
*r*		The reporting rate was assumed to have uniform distribution with a range between 50% and 80%.				
*s*		If a country did not report a slide positivity rate, values of *s* from other countries in the relevant WHO region were applied and assumed to occur with equal probability.				
*p* and *n*		If a relevant household survey was not available for a country, values of *p* and *n* from other countries in the relevant WHO region were applied and assumed to occur with equal probability.				

#### Method 2: Estimates from parasite surveys and risk maps

This method was used for 34 countries in sub-Saharan Africa where transmission is relatively homogenous and a broad categorization of malaria risk into either low transmission or high transmission is possible.

The annual incidence of malaria was estimated in two steps. First, populations in each country were classified as living at either high, low, or no risk of malaria. Malaria risk for each African country was defined according to climatic suitability, as per the Mapping Malaria Risk in Africa (MARA) project estimate for the year 2002 [2],[3]. The proportion of a country's 2002 population reported to be living at high, low, and no risk, was applied to the 2009 country populations as projected by the United Nations Population Division [10]. Second, incidence rates were derived for populations at high and low transmission risk from a review of longitudinal studies carried out in populations without malaria control activities, and these rates were applied to the number of people living in each risk group (Table 2) [2],[3],[11].

**Table 2:** Malaria case-incidence rates by transmission risk category (cases per 1,000 per year) used in estimating the number of cases by Method 2.

Age	High Transmission			Low Transmission			Southern Africa		
	*n*	*Median*	*IQR*	*n*	*Median*	*IQR*	*n*	*Median*	*IQR*
Under-5s	28	1.424	0.838–2.167	4	0.182	0.125–0.216	5	0.029	0.097–0.129
5–14 y	19	0.587	0.383–0.977	**—**	0.182^a^	0.125–0.216	**—**	0.029	0.097–0.129
≥15 y	7	0.107	0.074–0.138	**—**	0.091^b^	0.063–0.108	**—**	0.029	0.097–0.129
*Urban*									
Under-5s	**—**	0.712^c^	0.419–1.084	**—**	0.182^d^	0.125–0.216	**—**	0.029	0.097–0.129
5–14 y	**—**	0.587^d^	0.383–0.977	**—**	0.182^d^	0.125–0.216	**—**	0.029	0.097–0.129
≥15 y	**—**	0.107^d^	0.074–0.138	**—**	0.091^d^	0.063–0.108	**—**	0.029	0.097–0.129

a No observations available so assumed to be the same as that measured in children under 5 by Snow et al [3].

b No observations available so assumed to be half the rate of children 5–14 y by Snow et al [3].

c Estimated to be approximately half the rate of rural areas by Korenromp [11] and Carneiro et al [21].

d Considered to be the same as in rural areas by Korenromp [11].

IQR, interquartile range.

Because the incidence estimates were for 2002 populations or earlier, and those populations were not subject to malaria control measures, the estimates are adjusted downward for each country according to the expected impact of insecticide treated mosquito nets (ITNs) by 2009, and also to take account of lower incidence rates in urban areas [11].

#### Cases due to *P. falciparum*

For both methods an estimate of *P. falciparum* cases in each country was made by multiplying the total number of estimated cases by the percentage of cases that were found to be due to infection with *P. falciparum* in blood slide examinations that were carried out by national malaria control programs. The resulting estimate of the number of cases due to *P. falciparum* assumes that the species composition of cases attending public health facilities reflects that of all cases in the community.

#### Uncertainty analysis

An underlying distribution was assumed for each of the parameters used in incidence estimation (Table 1). Palisade@Risk (version 5.0) was used to sample from the distributions assumed for each parameter and each country. Latin Hypercube sampling without replacement was carried out using a pseudorandom number generator (Mersenne twister). For each country, we performed 1,000 calculations to yield a plausible distribution for the annual incidence of malaria cases, summarized with the mean, and bounded by 5th and 95th centiles.

## Results

### Malaria Incidence in 2009

**Figure 2:**
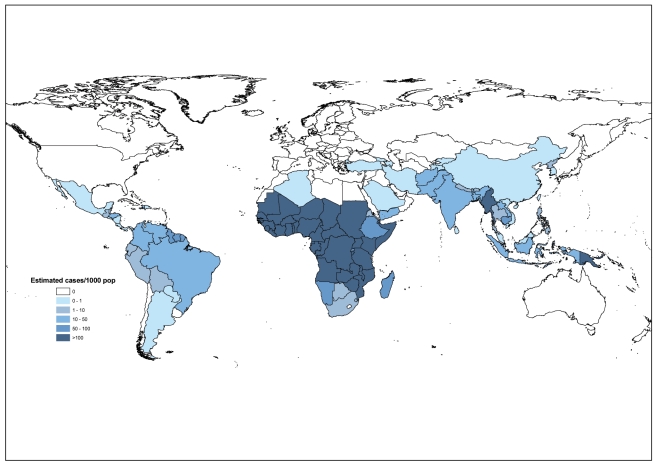
Estimated number of malaria cases per 1,000 population in 99 endemic countries made by method 1 (56 non-African and nine African countries) and method 2 (34 African countries).

Methods 1 and 2 applied to 99 countries together produced a total estimate of 225 million malaria cases worldwide in 2009 (5th–95th centiles, 146–315 million) (Table 3). The majority of cases (78%) were in the WHO African region, followed by the Southeast Asia (15%) and Eastern Mediterranean regions (5%, Figure 2). In Africa, there were 214 (133–302) estimated cases per 1,000 population, compared with 23 (17–34) estimated cases per 1,000 in the Eastern Mediterranean region and 19 (14–26) estimated cases per 1,000 in the Southeast Asia region (Table 3). Sixteen countries accounted for 80% of all estimated cases globally, all of them in the African region except for India and Myanmar (Text S1). The adjustments for malaria control measures and urban–rural differences reduced the estimated number of cases by 21% in Africa in 2009.

**Table 3:** Estimated number of all malaria cases in 2009 and the percentage of estimated cases that were due to infection with *P. falciparum*.

WHO Regions	Population (m)	Estimated Cases						*P. falciparum* (%)
		Best (000s)	Low (000s)	High (000s)	Best (per 1,000)	Low (per 1,000)	High (per 1,000)	
Africa	821	175,969	109,591	248,178	214	133	302	98
Americas	543	1,132	923	1,439	2	2	3	38
Eastern Mediterranean	523	12,120	8,668	17,816	23	17	34	84
Europe	272	0.64	0.54	0.76	0.0024	0.0020	0.0028	21
Southeast Asia	1,783	33,817	24,993	45,903	19	14	26	58
Western Pacific	1,638	2,257	1,910	2,618	1	1	2	79
World (99 countries)	5,580	225,296	146,085	315,955	40	26	57	91

**Figure 3:**
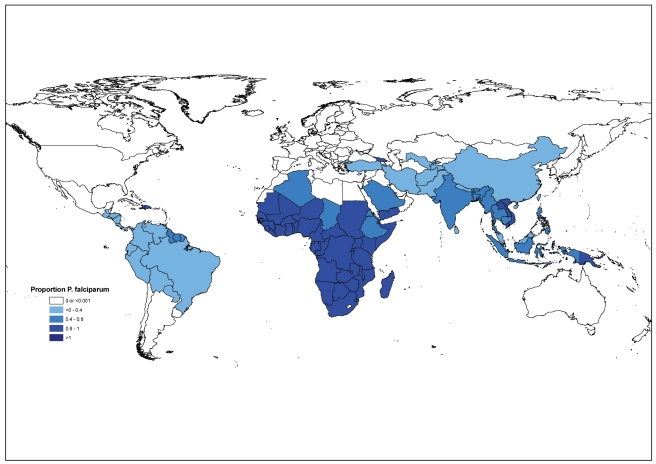
The percentage of reported malaria cases due to *P. falciparum* in 99 endemic countries.

An estimated 91% or 205 million cases were due to *P. falciparum* in 2009; 98% of estimated cases were due to *P. falciparum* in Africa and 65% of estimated cases were due to *P. falciparum* in other regions (Figure 3, Table 3). The percentage of estimated cases due to *P. falciparum* exceeded 75% in all but three countries in the African region (Algeria, Eritrea, and Ethiopia), but in only 11 out of 56 countries outside Africa.

Despite the differences between methods 1 and 2, the ratio of 95th/5th centiles for country estimates was approximately the same (geometric mean 2.3 for method 1 and 2.2 for method 2).

### Proportion of Cases Detected in 2009

Methods 1 and 2, together with national case reports, also yield estimates of the percentage of cases detected and confirmed by malaria control programs. We estimate that 8% of *P. falciparum* cases were reported in 99 countries in 2009 (Table 4). These percentages were ≤10% in the African, Southeast Asia, Eastern Mediterranean, and Western Pacific regions and higher in the American and European regions (Tables 3 and 4).

**Table 4:** A comparison of estimates of *P. falciparum* malaria cases obtained in this study for 2009 and by the MAP project for 2007.

Regions	Reported *P. falciparum* Cases (000s)	WHO 2009		MAP 2007		
		Estimated *P. falciparum* cases (000s)	Reported/Estimated (%)^a^	Reported (000s)	Estimated *P. falciparum* Cases (000s)	Reported/Estimated (%)^b^
Africa	1,2799	172,975	7 (7)	71,611	260,994	5
Americas	145	426	34 (50)	788	3,047	5
Eastern Mediterranean	950	10,153	9 (8)	8,449	13,875	7
Europe	0	0	(96)^c^	1.44	0	—
Southeast Asia	1,518	19,588	8 (8)	3,784	154,057	1
Western Pacific	182	1,774	10 (11)	1,946	18,959	1
World (99 countries)	15,594	204,915	8 (8)	86,579	450,932	3

a Numbers in brackets are for *P.falciparum* and *P. vivax* combined.

b MAP estimates are compared with reported cases in 2009 because there has been an increase in case reporting since 2007.

c No cases of *P. falciparum* were reported in the European region in 2009.

The overall proportions of cases detected depend on each of the elements of Model 1, and there were differences among regions in the importance of each element, and in the availability of data in 2009 (Table 5; Text S1). Confirmatory diagnostic tests (blood slides or RDTs) were used infrequently in Africa (34% of suspected cases in countries for which we applied method 1) as compared with other regions (94%, or 82% excluding India). Where diagnostic tests were done, the percentage positive (*s*) was less than 50% in all regions and almost all countries, suggesting that there is considerable overdiagnosis of malaria where slide-examination rates are low. Reporting was most complete in the European region (*r* = 96%) while information on reporting completeness was missing for eight out of 21 countries in the Americas. Southeast Asia had the lowest percentage of malaria patients that sought treatment in public health facilities (*p* = 14%); for other regions this percentage was at least 39%. Globally we estimate that 36% of malaria cases sought treatment in public sector facilities, while 42% sought treatment from private sector providers (including physicians, pharmacies, drug stores), and 22% did not seek treatment at all.

**Table 5:** Malaria cases reported to WHO in 2009, together with measures of parameters used to estimate incidence with model 1.

WHO Regions	*n* Countries Providing Data for Method 1	Suspected (000s)	Unconfirmed, *U* (000s)	Examined (Microscopy and RDT, 000s)	Examined/Suspected (%)^a^	Confirmed, *C* (Microscopy and RDT, 000s)	Confirmed/Examined (*s* %)^a^			Reporting Completeness (*r* %)^a^			Patients Seeking Treatment (1-*n* %)			Patients Treated in Public Health Facilities (*p* %)		
							Mean	Lower	Upper	Mean	Lower	Upper	Mean	Lower	Upper	Mean	Lower	Upper
Africa	9	86691	57293	29399	34	13165	23	4	53	80	72	87	63	58	68	41	36	46
Americas	21	6872	0	6872	100	562	6	3	12	77	72	82	80	65	95	69	54	84
Eastern Mediterranean	9	18082	6505	11578	64	1018	10	3	21	65	52	79	75	45	106	56	25	86
Europe	6	2207	0	2207	100	0.45	0	0	0	83	76	91	100	100	100	99	99	99
Southeast Asia	10	111105	643	110462	99	2404	17	5	36	78	71	85	62	52	71	17	7	26
Western Pacific	10	11703	1405	10297	88	247	21	8	42	87	87	87	71	56	87	40	24	56
World	65	236661	65845	170816	72	17396	12	8	30	78	75	81	71	64	77	50	44	57

**The data for each of the countries that reported, and for which method 1 was applied, are given in Text S1. Lower and upper estimates for parameters** ***r*** **, 1 −** ***n*** **, and** ***p*** **are 95% confidence limits.**

a Only considers countries for which method 1 was applied.

### Trends in Malaria Incidence, 2000–2009

The application of these methods for all years from 2000 to 2009 suggests that the number of cases increased worldwide until 2005 and has been falling slowly since then (Table 6, upper panel). The increase up to 2005 reflects low levels of intervention coverage in Africa and the effects of population growth. The number of cases per 1,000 population has been falling slowly in Africa and all other regions since 2000 (Table 6, lower panel). The estimated decline in cases per 1,000 population has been fastest in Europe and the Americas, and has accelerated globally since 2005 (0.5%/year 2000–2005, 3.2%/year 2005–2009), owing mainly to the steepening decline in Africa and the Americas.

**Table 6:** Trends in malaria incidence by WHO region and globally, 2000–2009, presented as total number of cases (millions, upper panel) and cases per 1,000 population (lower panel).

Cases	2000	2001	2002	2003	2004	2005	2006	2007	2008	2009	Decline Percent/Year
**Millions**											
Africa	173	178	181	185	187	188	187	186	181	176	0.2
Americas	2.8	2.3	2.2	2.1	1.9	1.9	1.7	1.5	1.1	1.1	−9.9
Eastern Mediterranean	15	16	17	16	14	12	12	12	13	12	−3.6
Europe	0.0	0.0	0.0	0.0	0.0	0.0	0.0	0.0	0.0	0.0	−49.6
Southeast Asia	38	38	35	35	37	39	34	32	34	34	−1.4
Western Pacific	2.8	2.5	2.2	2.5	2.8	2.3	2.5	2.1	1.9	2.3	−2.6
World	233	236	237	241	243	244	238	233	231	225	−0.4
**Per 1,000 population**											
Africa	264	263	262	261	258	253	245	237	225	214	−2.2
Americas	5.9	4.8	4.4	4.1	3.8	3.7	3.2	2.7	2.0	2.1	−11.2
Eastern Mediterranean	45	44	46	44	39	31	32	30	32	29	−5.8
Europe	0.41	0.29	0.23	0.18	0.11	0.06	0.03	0.02	0.01	0.01	−50.7
Southeast Asia	24	24	22	21	22	23	20	18	19	19	−2.9
Western Pacific	1.8	1.6	1.4	1.6	1.8	1.4	1.6	1.3	1.1	1.4	−3.4
World	50	50	49	49	49	48	47	45	44	42	−1.8

## Discussion

Our estimate of malaria case incidence for the African region is 176 (110–248) million cases in 2009 of which 173 million were estimated to be due to infection with *P. falciparum*. This estimate is lower than the most recent estimate from MAP of 261 (241–301) million *P. falciparum* cases in 2007 (Table 4) [4]. The latest MAP figures are part of a fluctuating series of global estimates from MAP and WHO: 214 million for year 2000 [3], 365 million for 2002 [5], and 226 million for 2004 [11]. The differences among these numbers are due primarily to changes in estimation methods, rather than to changes in malaria epidemiology.

Although our interpretation of method 2 and that of MAP are based on the same principles, MAP's estimates [4] are mostly higher for African countries than ours. We believe that the main reason for this is that we have allowed for the increasing coverage of insecticide-treated nets. Without ITNs, our estimate of incidence in Africa would be 226 (140–318) million cases, of which an estimated 222 million are due to *P. falciparum*, and our estimates would be even closer to MAP's estimated 261 million cases in 2007 if our estimates were based on the same data (MAP's full dataset is not yet publicly available). The bigger differences, however, are for non-African countries, where we have relied more on routine surveillance (estimating 49 million cases, of which 32 million are due to *P. falciparum*) and MAP has favoured surveys and risk maps (estimating 190 million *P. falciparum* cases) (Table 4). Our estimate of the number of cases due to malaria other than *P. falciparum* (20.3 million in Table 3, mainly *P. vivax*) is also less than found in some other studies [12],[13]. To resolve these discrepancies, we need to consider why estimates based on routine surveillance might be too low and why those based on surveys might be too high. The following two sections examine the strengths and weaknesses of both methods, as implemented in the present study and previously by MAP.

### Estimates Derived from Routine Case Reports

The potential weakness of surveillance-based estimates lies in the quality of the data that are used to measure five key variables: reporting completeness (*r*), the proportion of suspected malaria cases that is parasite-positive (*s*), the proportion of malaria cases that is due to each *Plasmodium* species, the extent to which patients seek treatment (1 − *n*), and whether patients use public sector health facilities (*p*).

Box 1 lists 12 possible types of error in measuring these variables and gives possible sources of systematic bias or random error. Some specific deficiencies in malaria surveillance data have become clear from the analyses carried out during this study, and there are broad regional patterns: in Africa, the small proportion of patients who receive a confirmed diagnosis by microscopy or RDT; in the Americas, the lack of information on the number of reports expected and received from health facilities; in the Eastern Mediterranean, the absence of data on the use of public and private health facilities; in Southeast Asia, the small fraction of cases captured by the public health reporting system. In all regions, there is a risk of underestimating untreated cases of malaria because mild fevers that might be due to malaria are missed in household surveys (*n* is too small and/or *p* is too large).

**Box 1. Potential Problems and Consequences of Uncertainty in Parameters Used to Estimate Malaria Cases by Method 1**

**Reporting Completeness**

*Problem 1.* Countries may not keep a complete and up-to-date list of all open health facilities, and reporting completeness may have been provided only for those facilities that are known to malaria control programmes.

*Consequence:* Reporting completeness overestimated and malaria burden underestimated.

*Problem 2.* If health facility reports are aggregated at a district level then, in the absence of other information, when a district report is received it may be assumed that all health facilities in the district have reported. Similarly if reports are aggregated quarterly they may contain incomplete monthly information but be counted as complete. If accurate monitoring of the percentage of reports received is not kept, then reporting completeness may be overestimated.

*Consequence:* Reporting completeness overestimated and malaria burden underestimated.

*Problem 3.* The analysis undertaken does not consider the type of institution failing to report. Failure of a hospital to report will generally have a greater influence on the reported number of malaria cases than a health post. In some countries malaria programmes have difficulty obtaining data from hospitals that use a separate reporting system. In other countries, missing reports may be mostly those from health posts and reporting completeness underestimated.

*Consequence:* If hospitals are more likely to underreport, the reporting completeness will be overestimated. If health posts are more likely to underreport, reporting completeness will be underestimated.

**Utilization of Public Health Facilities**

*Problem 4.* Demographic and Health Survey (DHS) and Multiple Indicator Cluster Survey (MICS) were used to estimate the proportion of malaria cases attending public health facilities, private health facilities, pharmacies or shops and those not seeking treatment at all. These proportions were derived from children under 5 who experienced fever in the 2 wk before the survey. Care-seeking behaviour in children under 5 seemed to provide a reasonable approximation to care-seeking behaviour in other age groups in two countries where it could be checked, but may not apply elsewhere.

*Consequence:* There is no comprehensive evidence to suggest that other age groups use health services more or less than children under 5 y in response to reported fever. Potential consequence unknown.

*Problem 5.* Care-seeking behaviour for self-reported fever may not necessarily reflect care-seeking behaviour for suspected or confirmed malaria.

*Consequence:* There is no comprehensive evidence that fever differs significantly from true malaria. Potential consequence unknown.

*Problem 6.* Only nine of the 69 household surveys analysed were conducted in 2006. 85% of surveys were from 2000 or later, with the median age of survey being 5 y. Utilization of health services may therefore be under- or overestimated.

*Consequence:* There is no evidence that the percentage of fever cases using government health services has either increased or decreased. Potential consequence unknown.

*Problem 7.* A single national estimate of the proportion of fever cases attending public health facilities was used. In some countries, the availability and accessibility of services may be greater in areas with less malaria. Conversely services may be less accessible in areas where there is more malaria.

*Consequence:* Potential overestimation of the proportion of malaria cases attending public health facilities. Simultaneously the proportion of malaria cases using private health facilities may be overestimated. The combined effect of these tendencies is unknown.

*Problem 8.* The uncertainty analysis considered only sampling variation in the estimation of *u* and *n*. The potential effect of misclassification of treatment outlets as being covered by the Health Management Information System (HMIS) or not was not explored.

*Consequence:* Potential underestimation of the uncertainty regarding case estimates.

**Slide Positivity Rate**

*Problem 9.* Health facilities that undertake slide examination may only do so for selected patients, e.g., those admitted, or for adults.

*Consequence:* If slide examination is reserved for more severe cases, the number of confirmed malaria cases may be overestimated. If slide examination is reserved for adults, the number of confirmed malaria cases may be underestimated. The combined effect of these tendencies is unknown.

*Problem 10.* A slide positivity rate (SPR) derived from selected government facilities is applied to suspected malaria cases attending other facilities to estimate confirmed malaria cases. Health facilities not undertaking case confirmation may differ qualitatively from those undertaking slide examination (e.g., they may be in different parts of the country) and obtain a different SPR.

*Consequence:* If facilities undertaking slide examination are located in more developed or urban areas, the true proportion of suspected cases that are confirmed may be underestimated. If slide examination is more likely to be undertaken in areas where malaria transmission is more intense, the proportion of all cases that are confirmed will be overestimated. The combined effect of these tendencies is unknown.

*Problem 11.* A SPR from public health facilities is applied to private facilities including shops and pharmacies, but the true rate may be different.

*Consequence:* No evidence that slide positivity in the private sector differs from that in the public sector. Potential consequence unknown.

*Problem 12.* On average a SPR of half of that found in public health facilities is applied to fever cases not attending facilities; the range of SPR used being from 0 to *s*.

*Consequence:* Knowledge of infection rates in fever cases that do not seek treatment is insufficient. Potential consequence unknown.

As weaknesses in surveillance are recognized and addressed, estimates will be improved, and the ability of national control programs to monitor progress and manage resources will be strengthened. In Southeast Asia, for example, it is clear that national malaria control programs need to work more closely with private providers to ensure appropriate diagnosis and treatment and accurate monitoring. Diagnostic accuracy will improve as parasitological diagnosis of malaria, including use of RDTs, is made more widely available and malaria control programs follow international guidance [14],. Routine malaria surveillance should also serve to reinforce the monitoring and evaluation of other major diseases including acute respiratory illnesses, diarrhoeal diseases, and tuberculosis.

There is also scope to improve the design and coverage of household surveys in order to assist the interpretation of surveillance data [16]. Data collected on outpatient and inpatient attendance rates measured in populations, which can be compared to the same rates measured from the Health Management Information System (HMIS), help to assess the completeness of health facility reporting. We also need to ascertain why fever cases do not attend health facilities, for example is it because fevers are mild, or because facilities are geographically inaccessible or because services are too costly to use (travel costs, fees for users, and so on). Such information is seldom included in Demographic and Health Survey (DHS), Multiple Indicator Cluster Survey (MICS), and Malaria Indicator Surveys though it is sometimes available in broader health or socio-economic surveys.

In sum, estimates based on surveillance data might be too low or too high. Having made a checklist of the potential sources of bias (Box 1), detailed investigations are needed to identify the source and magnitude of error for each country and measures undertaken to address deficiencies identified. New guidance on strengthening surveillance systems for malaria control in different epidemiological settings and elimination will be published by WHO during 2012.

### Estimates Derived from Surveys and Risk Maps

There are two reasons prima facie why the higher estimates of case incidence derived from surveys [4] might be too high, especially for non-African countries. First, the MAP estimates (based in part on risk maps) imply that surveillance misses a large fraction of cases, even in countries that have strong health information systems, and where a relatively high proportion of cases has signs and symptoms. MAP estimates suggest that the percentage of cases detected by surveillance was similar for the Americas and Africa (5%) but, unexpectedly, lower in the Southeast Asia (1%) and Western Pacific regions (1%) than in Africa, for the countries that provided data (Table 4). These detection rates in Asia are far lower than those reported in seven specific studies on detection cited by MAP [4], which were in the range 17%–37% (except for India, 2%–11%).

The details of case reporting from specific countries support the view that MAP estimates are too high. The Vector Borne Disease Control Programme (VBDCP) in India examined blood slides from 95.4 million suspected cases in 2009, approximately 8% of the population, yet detected only 844,000 slides positive for *P. falciparum* (0.9%). If the slide positivity rate is correct, then India would have to examine 11.3 billion samples from suspected malaria cases annually to find MAP's estimated 102 *P. falciparum* million cases. This is 9.4 suspected cases per person nationwide. By the same logic, the number of suspected cases that need to be examined to obtain MAP estimates in other highly endemic Asian countries are 9.5 in Viet Nam, 8.5 in Thailand, and 5.2 in Malaysia. These ratios would be higher still if we considered only the subpopulations at malaria risk.

Second, MAP's estimates of *P. falciparum* case incidence for many non-African countries are as high as those in Africa. Thus Myanmar (527/1,000) has a higher estimated incidence rate than Benin, Nigeria, and Togo (484–491); Laos is as high as Senegal and Chad (all 278); and Malaysia (64) and Philippines (63) are similar to Ethiopia (72). Comparable incidence rates in these African and non-African countries seem unlikely not least because a review of longitudinal studies undertaken by the MAP team indicates lower malaria incidence rates in non-African settings than in Africa [8].

There are three further aspects of method 2 that could overestimate incidence, either in our hands or with the more sophisticated approaches now used by MAP. First, the surveys of parasite prevalence and case incidence that determine the spatial distribution of malaria risk vary in method and purpose. The surveys were not designed to give unbiased estimates of the national prevalence of malaria infection. One potential problem is that parasite prevalence surveys have been carried out in areas of relatively high malaria incidence. In India, for example, most surveys have been done in the high incidence areas of Assam and Orissa [17]. Extrapolating from these sites to populations in different areas that are at lower risk would lead to overestimates of case incidence. Overestimates could also arise if surveys were preferentially done in rural areas where malaria incidence is typically higher than in urban areas.

The second problem, related to the issues surrounding prevalence surveys, is that the procedure for delineating areas with stable malaria tends to overestimate the population at risk where the administrative unit is large. For China, MAP classified populations at the second administrative level (prefecture), noting whether the number of reported *P. falciparum* cases was more than 1 per 10,000 [6]. Prefectures in Yunnan and Hainan provinces have a median population size of 2.4 million, and yield a population at stable risk of malaria (>0.1 case/1,000 population/year) of 9.3 million. However, if the assessment is done at county level (median population 280,000), only 3.7 million would be classified as living in areas with stable malaria, a 2.5-fold difference.

Third, the latest risk map from MAP is intended to represent the situation in 2007 [18], but many of the constituent surveys of parasite prevalence are much older. Of the 7,953 surveys used by MAP, 41% were done before 2000 [7]. To define the relation between parasite prevalence and malaria incidence in the African region, MAP used 25 African surveys (from just six countries), 16 of which were started before 2000 [8]. For non-African countries, there were 116 surveys, 80 of which were started before 2000.

In addition to these possible sources of bias, other factors affect uncertainty (Box 2). For example, our use of only two risk categories is obviously a coarse classification that MAP has refined. MAP has defined the statistical relation between parasite prevalence and malaria incidence using Bayesian methods in order to make best use of prior and posterior information. However, most of the data points (which constitute the posterior distribution) that contribute to this analysis lie outside the range of the 95% credible relationship between incidence and prevalence [8]. That is, the relation between incidence and prevalence is highly variable. The implication is that the data have little influence on the derived relationship malaria incidence and parasite prevalence, which is strongly determined by prior assumptions. Consequently, it is not clear for which countries the MAP statistical model has under- or overestimated incidence.

**Box 2. Potential Problems and Consequences of Uncertainty in Parameters Used to Estimate Malaria Cases by Method 2**

*Problem 1.* The delimitation of only two risk categories (high and low) does not provide for a fine categorization of malaria risk.

*Consequence:* A particular risk category may contain a wide range of malaria incidence and death rates.

*Problem 2.* The model to determine the suitability of the climate model for malaria transmission was based on a 30-y average of climatic variables.

*Consequence:* There is known to be variation year by year in the suitability of climate for malaria transmission, and this annual variation was not taken into account in the uncertainty analysis, nor was the suitability of the climate for malaria transmission estimated for specific years.

*Problem 3.* The studies used to derive basic incidence rates were not designed to be representative of the levels of endemicity they purport to describe, are small in number, and show a wide variation in measured case incidence with few, if any, studies in urban areas and low-risk rural areas which required rates to be inferred.

*Consequence:* If surveys have been done in areas of relatively high incidence, extrapolating from these sites to populations at lower risk would lead to overestimates of case incidence.

*Problem 4.* The studies used to derive basic incidence rates within categories of endemicity, urbanicity, and age group were mostly conducted during the 1990s and earlier when treatment was often given presumptively in highly endemic areas (perhaps reducing the incidence of recurrent malaria), when the malaria case definition may have differed from that used in this study, and when incidence rates within endemicity categories may not be the same as those between 2000 and 2009. Notably, the influence of artemisinin-based treatments and ITNs on reducing transmission and case rates was not captured.

*Consequence:* Current incidence rates might be overestimated (but possibly underestimated) by using historical data.

*Problem 5.* The adjustments made to take into account the effects of interventions on case incidence are based on a relatively small number of clinical trials, run for only 2 y after the introduction of ITNs, which tended to show higher levels of intervention coverage than observed in most countries. Moreover the assumption of efficacy varying linearly from lower to higher coverage levels was not based on empirical evidence.

*Consequence:* Adjustments based on these trials may give an optimistic view of the reduction incidence, and therefore give incidence estimates that are too low.

Finally, our assessment of malaria trends in Africa, which takes into account only only the impact of ITNs and not other control measures, might underestimate the rate of decline. We have not taken into account the use of indoor residual spraying or the availability of more effective treatment with ACTs. Moreover, it is well known that factors other than vector control influence mosquito abundance, species composition, and human biting rates. These factors include urbanization, trends in rainfall, temperature, and humidity, changes in land use, and improved housing construction [19]. To draw a bigger picture of malaria trends by region and globally requires longer and more reliable time series of data from a larger number of countries than currently available. Time trends data are especially limited in the African region [1],[20].

### Conclusion

Method 1, based on routine surveillance data, gives lower estimates of case incidence than method 2, based on population surveys, especially for non-African countries. The large discrepancies for some non-African countries, notably India, will only be resolved with further data and careful validation.

Although the best assessment of malaria burden and trends today must rely on a combination of surveillance and survey data, accurate surveillance is the ultimate goal for malaria control programs (expanding the database depicted in Figure 1). Routine surveillance has two particular advantages for estimating case incidence, spatially and through time. First, data compiled annually allow for the effects of changes in the array of factors that influence case incidence from place to place (at the level of provinces, counties, etc.) and from year to year, especially the factors linked to climatic variation and malaria control interventions. And the assessment of incidence trends over time is likely to be more accurate than the assessment of absolute incidence. By contrast, population surveys cannot be done annually and are costly when designed to cover whole countries with large enough samples to detect spatial variation, particularly when parasite prevalence is low. Second, annual monitoring is an essential part of running effective control programs, tying budgets and expenditures to the distribution of commodities and to clinical and epidemiological outcomes. To strengthen surveillance requires a critical evaluation of all the types of error we have identified in this paper. Only with investigations of this kind can we confidently assess malaria burden and trends, and the return on investments in control programs.

## Supporting Information

Text S1 **Supporting information on methods for estimating malaria incidence.**
(DOC)

## Abbreviations

ITN: insecticide treated mosquito net
MAP: Malaria Atlas Project
RDT: rapid diagnostic test
WHO: World Health Organization
