# Supplementary material for: Worldwide Incidence of Malaria in 2009: Estimates, Time Trends, and a Critique of Methods
Source: PLoS Med. 2011 Dec 20;8(12):e1001142. doi: 10.1371/journal.pmed.1001142 (PMC3243721; doi:10.1371/journal.pmed.1001142)
Supplement: Text S1 — Supporting information on methods for estimating malaria incidence. (DOC) [file pmed.1001142.s001.doc]

# Worldwide Incidence of Malaria: Estimates, Time Trends and a Critique of Methods

**Supporting Information (Text S1)**

Richard Cibulskis, Maru Aregawi, Ryan Williams, Mac Otten, Christopher Dye

This annex gives a fuller account of the two methods for estimating malaria incidence, based on routine case reports (Method 1) and parasite surveys and risk maps (Method 2).

The two methods for estimating malaria incidence are:

Method 1 adjusts the reported malaria cases for reporting completeness, the extent of health service utilization and the likelihood that cases are parasite-positive. Where data permit, this is generally the preferred method and it was used for countries outside Africa and selected African countries.

Method 2 uses an empirical relationship between measures of malaria transmission risk and case incidence; this procedure was used for countries in Africa where data from surveillance systems was not reliably reported.

**Method 1: Estimates from routine case reports (surveillance)**

***Basic Procedure*** The principal source of data was outpatients attending health facilities as recorded in the health management information system (HMIS) of a Ministry of Health, or by disease surveillance systems. National malaria control programmes (NMCP) were asked to report on: (1) the number of slide or rapid diagnostic test (RDT) examinations undertaken; (2) the number of cases confirmed by slide examination or Rapid Diagnostic Test (RDT); and (3) the number of unconfirmed or probable cases recorded – these are cases that were treated as malaria but not tested or confirmed. The diagnostic algorithm for malaria is in Figure S1.

Figure S1. Diagnostic algorithm for malaria cases.

The following steps were used to estimate the number of malaria cases in a country:

1. The total number of parasite-positive cases that attended health facilities covered by the HMIS of a ministry of health was estimated; this is given by the confirmed malaria cases plus the unconfirmed cases multiplied by the slide positivity rate.
2. The revised estimate of confirmed cases was adjusted to take account of missing HMIS reports; by dividing by the health-facility reporting completeness fraction.
3. The estimated number of confirmed cases attending health facilities in the HMIS was then adjusted to take into account the propensity of fever cases to use health facilities not covered by the HMIS (e.g. those going to the private sector), or not to seek treatment at all.

Upper and lower limits for the estimated number of cases, *M*, arising in any given year in a country are given by:

$$M_{upper}=\frac{C+sU}{rp}$$

$$M_{lower}=\frac{(C+sU)(1-n)}{rp}$$

Where:

*C* = Reported number of confirmed malaria cases in a year.

*U* = Reported number of unconfirmed cases in a year – cases suspected of being malaria but not tested or confirmed, sometimes known as probable cases.

*s* = The proportion of slides examined that are positive for malaria parasites, often known as the slide positivity rate (SPR). In countries that are unable to undertake microscopic examination of all suspected cases, *s* was derived from a selection of facilities, or treatment outlets, that undertake case confirmation. The proportion of cases found to be positive, *s*, was applied to cases that were not confirmed. Cases may be diagnosed microscopically or with RDTs. The sensitivity and specificity of RDTs for detecting clinical *P. falciparum* malaria (symptoms suggestive of malaria plus *P. falciparum* parasitaemia detectable by microscopy) in persons living in malaria endemic areas who present to ambulatory healthcare facilities with symptoms of malaria is typically >95% [1]. However, the use of RDTs was not widespread up to 2009, the year in which data were collected for this study. ,

*r* = Completeness of health-facility reports. Typically this is the number of outpatient health-facility reports received divided by the number of facility reports expected. The expected number of reports is given by the number of health facilities multiplied by the number of reports expected to be submitted by each health facility in a year, which is 12 for a monthly reporting system. The quantity *r* has been reported by countries as lying within three possible ranges (less than 50%, 50–80% and > 80%). In order to calculate an average reporting rate, it has been assumed that the reporting rate has a triangular distribution in the outer ranges and a uniform distribution in the middle with expected values in these ranges of 33%, 65% and 87% respectively.

Figure S2. Variation in utilization of health facilities for treatment of fever by age in (A) India and (B) Indonesia. Analyses of household surveys undertaken in 2004 in India [2] and in Indonesia [3] suggest that the percentage of children under 5 using health facilities for treatment of fever provides a good approximation to the percentage of older age groups using health facilities for treatment of fever.

*p* = The proportion of the population with fever (or suspected malaria) that seeks treatment from health facilities covered by the public reporting system. This was derived from household survey information on whether or not children under 5 years, with fever in the previous two weeks, sought treatment and where. The household survey used for most countries was a Demographic and Health Survey (DHS) [4] or Multiple Indicator Cluster Survey (MICS) [5] that only considers fever in children under 5. Fever treatment rates for older age groups are assumed to be the same as those for children under 5. Household surveys undertaken in Indonesia (Susenas) [3] and India (NSSO) [2] suggest that this assumption is reasonable (Figure S2).

*n* = The proportion of fever cases (or suspected malaria) that does not seek treatment. This was derived from household survey information on whether or not treatment for fever was sought, as described above.

If there was only one household survey for a country then values of *p* and *n* were used for all years for which estimates were made. In general, there has been little change in the proportion of fever cases seeking treatment over time (Figure S3), hence results from one year can provide a reasonable approximation to the proportion of fever cases seeking treatment in another year, unless there is specific knowledge that treatment-seeking behaviour has changed.

For countries with more than one survey the percentage of fever cases seeking treatment from public or private sector facilities was interpolated by linear regression for years in between surveys and assumed to remain constant after the latest survey and before the earliest survey.

Figure S3. Variation over time in the percentage of fever cases seeking treatment at health facilities. Of 58 survey differences that could be compared over time using DHS and MICS data sets, the median annual change in the proportion of febrile children attending health facilities was 0.1% (inter-quartile range: 1.4%–2.0%).

For DHS surveys, the measure of *p* is the proportion of fever cases seeking treatment from hospitals, health centres and health posts run by the government and missions but excludes cases treated at private for-profit organizations and pharmacies or shops. It also excludes those cases not seeking any treatment. In some cases the proportion attending public health facilities was adjusted to take into account specific information about malaria treatment seeking behavior. For example, in some countries antimalarial medicines are not available through private sector providers so use of private providers was assumed to be zero and patient attendances were assigned to public health facilities. If more than one source of treatment was used, the source of treatment considered to be at the highest level or furthest along the referral chain was selected, with government facilities ranked higher than mission or private facilities as in the following order: government hospital, government health centre, government health post, other government facility, mission hospital, mission health centre, mission health post, private clinic or health centre, private doctor, private other, community health worker, pharmacy, shop, traditional, other. The ranking was intended to reflect the probability that a case attending multiple treatment outlets would be recorded in the HMIS of a ministry of health, but the precise classification used has little effect on the final results since the percentage of children using more than one outlet for treatment is only 5% (Figure S4).

The majority of MICS surveys record the proportion of fever cases seeking treatment at health facilities but the type of health facility attended, and whether it is in the public or private sector or not, is not distinguished. Hence for MICS surveys, information on health-facility utilization for acute respiratory infection (ARI) was combined with fever treatment to estimate the proportion of fever cases attending public-sector facilities (*p*) as below:

$$p=h_{fever}\times\frac{g_{ARI}}{h_{ARI}}$$

Where:

*h_fever_* = The proportion of children with fever in the past two weeks who attended a health facility.

*h _ARI_* = The proportion of children with both cough and rapid breathing in the past two weeks who attended any health facility.

*g_ARI_* = The proportion of children with both cough and rapid breathing in the past two weeks who attended a government or mission health facility.

For countries with both a DHS and a MICS survey, the measurements of *p* were correlated, but DHS tended to be lower than MICS (Figure S5). Nevertheless, we used either where available. For Papua New Guinea and Myanmar the household surveys, a DHS and a MICS respectively, did not record health-facility utilization for fever. Hence information on health-facility utilization for ARI (cough and/or rapid breathing) was used to estimate *p* since it is observed for other countries that health-facility utilization rates for ARI serves as a reasonable approximation for health-facility utilization rates for fever (Figure S6).

For the Solomon Islands and Guinea-Bissau, confidence intervals of *p* could not be calculated because of insufficient information on the sampling procedures employed. Confidence intervals were therefore assumed to lie 7.5 percentage points either side of the point estimate of *p,* since 90% of measured confidence intervals were of this size or smaller in other countries.

Figure S4. Percentage of fever cases using one or more outlet for treatment. In an analysis of 105 DHS surveys (with each survey weighted equally), the proportion of fever cases using more than one treatment outlet was only 5%.

Figure S5. Percentage of fever cases taken to health facilities. Comparison of estimates of *p* (the percentage of fever cases taken to a facility included in a country’s HMIS) derived from MICS and DHS show that they are correlated (*r^2^* = 0.45) but that DHS measurements are somewhat lower than MICS.

Figure S6. Percentage of fever cases seeking treatment for fever (at all outlets) plotted against the percentage of ARI cases seeking treatment. Analysis of 105 demographic and health surveys indicates that the percentage of children with ARI that seek treatment can provide a good approximation to the percentage of children with fever that seek treatment. The two measures are highly correlated (*r^2^* = 0.94), though the percentage of children seeking care for fever is a little higher than those seeking care for ARI (*y* = 0.05 + 0.97*x*).

The difference between upper and lower limits on *M* reflects the extent to which malaria cases are treated in the health system (both formal and informal). The upper limit produces an estimate of the number of malaria cases if all fever cases were equally likely to be malaria whether they sought treatment or not, i.e. those that do not seek treatment have the same slide positivity rate, *s*, as those that do. The lower limit estimates the number of malaria cases if only those fever cases that seek treatment were malaria, i.e. the slide positivity rate, *s*, of fever cases not seeking treatment is zero. In practice the true value will probably lie between these points. It will lie close to the lower limit in areas where accessibility to services is good and all cases that need treatment actually seek it. It will lie closer to the upper limit in areas where accessibility of services is poor, and many malaria cases go untreated. It was only possible for a few countries to assume that all cases seek treatment; these were countries in Europe and some countries nearing malaria elimination (where most infections produce symptomatic cases and where access to services is good). In the absence of detailed information on the structure of health services in a country, it is expedient to derive a single point estimate, *M* from the arithmetic of average of *M_lower_* and *M_upper_*.

Values of *C*, *U*, *s*, *r*, *p*, and *n* are shown for each country in Table S2.

***Missing data*** The formulae used to estimate the number of malaria cases require six parameters. Four parameters are obtained from the national malaria control programmes (*P, C, r, s*) and two from household surveys (*n* and *u*). Some countries did not provide values for all parameters or a household survey may not have been available for analysis. In such cases, values for the parameters were imputed according to the scheme below:

*U* or *C* If *U* or *C* were missing for a year, or data were considered to be unreliable (e.g. because they were radically different from other years), the value of U or C was sampled from other years for which data were considered reliable with each year weighted equally. For 2009, 3 countries did not provide data on U or C (Algeria, Argentina and French Guiana) while 3 countries provided data that were inconsistent with previous years (Indonesia, Somalia and Yemen).

*r* If the reporting completeness, *r,* was not reported for a single year the most frequent value for the country between the years 2000 and 2009 was chosen or, exceptionally, a rate that was considered more consistent with the reported number of cases. If *r* was missing for all years, it was assumed to lie between 50% and 80% with a point estimate of 65%.

*s* If the slide positivity rate, *s,* was missing for a year, then it was sampled from years for which data were available with each year weighted equally. If *s* was missing for all years, *s* was sampled from other countries in the region with each country weighted equally.

*p, n* If *p* and *n* were missing, household surveys from other countries in the region were used to derive regional averages of *p* and *n* with each country weighted equally. For some countries approaching elimination or for those where it is known that almost all cases use public sector facilities, *n* was assumed to be 1% and *p* to be 98%.

***Uncertainty analysis*** An attempt was made to quantify the uncertainty in each of the parameters and to use this information to construct a plausible range within which it is reasonably certain that the estimate of the number of fever and malaria cases lies. The underlying distribution assumed for each of the parameters is shown in Table 1 of the main text. Palisade @Risk software (version 5.0) was used to sample from the distributions assumed for each parameter and each country. Latin Hypercube sampling was performed 1000 times to yield a distribution for the estimated number of fever and malaria cases for a country, from which the mean value was taken together with 5% and 95% uncertainty limits for the mean.

***Limitations*** Not all aspects of the uncertainty were modeled. Moreover, the assumptions regarding the distribution of some parameters may not always apply. Particular concerns regarding different parameters are summarized in Box 1 of the main text. Recognition of the limitations, along with information on how data are collected in a country, suggests steps that may be taken to improve estimates in particular countries. An index of the quality of data available in each country has been constructed taking into account the completeness of data in the HMIS of a ministry of health and the availability of household survey information. It ranges from zero (no data) to 10 (maximum score). Values are summarized regionally in Figure S6. Where data systems are good and household surveys recent, greater confidence may generally be placed in an estimate.

**Method 2: Estimates derived from surveys and risk maps**

***Basic Procedure*** The procedure follows that of Snow et al [6] and Korenromp et al [7]. The number of malaria cases was estimated in two steps: firstly, populations in each sub-Saharan country were classified as living at either high, low or no risk of malaria; secondly, high, low or zero case-incidence rates were applied to the number of people living in each endemicity class as derived from literature reviews. The case-incidence rates were subsequently adjusted to take into account the level of malaria intervention coverage (use of ITNs and IRS only). Such an approach was attempted only (i) for countries in sub-Saharan Africa where it is assumed that transmission is relatively homogenous and a broad categorization of malaria risk into low-transmission and high transmission is possible; and (ii) for countries with profound deficiencies in malaria reporting.

***Defining malaria risk*** Malaria risk for countries in sub-Saharan Africa was defined according to climatic suitability, as per the Mapping Malaria Risk in Africa (MARA) project estimate for the year 2002 [8]. Long-term climate data were used to define the probability of malaria transmission, in terms of a “climate suitability index”, ranging from 0 (unsuitable) to 1 (very suitable), at a resolution of 5 × 5 km [6]. Areas with a MARA index greater than 0.75 were considered to be at high transmission risk, and areas with a MARA index below 0.75 at low transmission risk [8,9]. These two categories correspond broadly with parasite prevalence in childhood populations greater than or equal to 25%, and less than 25% respectively [10]. The proportion of a country’s 2002 population reported to be living at high, low and no risk, as shown in Table S1, was applied to the 2006 country populations as projected by the United Nations Population Division [11,12].

***Deriving case-incidence rates*** "Basic malaria incidence rates" were derived for populations at high and low transmission risk from a review of longitudinal studies of populations not benefiting from malaria prevention [6]. More than 20 studies were from high-intensity transmission areas (defined as parasite prevalence ≥ 25% or MARA climate suitability index ≥ 0.75), and four from low-intensity transmission areas (parasite prevalence < 25% and MARA climate suitability index between 0 and 0.75). The rates for southern African countries were derived from national surveillance data from Botswana, Namibia, South Africa, Swaziland and Zimbabwe, assumed to be independent of age. The number of studies and median incidence rates with inter-quartile ranges (IQR) for each category of transmission risk are shown in Table 2 of the main text.

***Adjusting for urban–rural differences*** Adjustments to basic incidence rates were made for urban settings following Korenromp [7]. Urban incidence rates for children under 5 were assumed to be half those of rural areas, as determined (approximately) from other community-based studies of urban*–*rural differences [13]. Incidence rates for other age groups in high-transmission areas were considered to be the same as for rural areas. Incidence rates for urban low-transmission areas were considered to be the same as rural low-transmission areas, although Carneiro et al [13] note one study in Kenya which estimated urban incidence rates to be 40% of those in rural areas for children under 5. Similarly, Korenromp [7] notes that, outside Africa, urban incidence rates were approximately one third of those in rural areas. Hence the assumption that urban incidence rates in low-transmission areas are the same as rural incidence rates may lead to an overestimate of malaria incidence in urban low-transmission areas. However, the influence on individual country estimates is relatively minor as less than 25% of any country is considered to be urban and low-transmission, while for sub-Saharan Africa as a whole it is about 6.5%.

***Adjusting for malaria preventive activities***  Since the basic incidence estimates were for populations not subject to malaria preventive measures, they were adjusted downward for each country according to the expected impact of ITNs (they were not adjusted for indoor residual spraying or drug use). The approach used was similar to that adopted for malaria mortality [14] Malaria incidence rates were reduced linearly by 0.5% for each 1% increase in the percentage of households owning at least one ITN in accordance with observations in randomized controlled trials [15] and similar to the 0.55% reduction per 1% increase used in the LiST model of child survival [16]. The percentage of households owning at least one ITN was derived from a model that takes into account available household survey data and, for years in which a survey was not conducted, estimates coverage based on the number of ITNs delivered by manufacturers and distributed by NMCPs [17]. The rates used for the percentage of households owning at least one ITN are those presented in the World Malaria Report 2010 [18]. The potential effect of IRS was not incorporated owing to less certainty about the magnitude of the effective of the intervention on malaria morbidity and because of the unknown extent of overlap in populations protected by indoor residual spraying and ITNs. Similarly the potential effect of the increased availability of artemisinin combination therapies for the treatment of *P. falciparum* is not taken into account because of uncertainty over the effect it has on malaria morbidity rates in populations. This is likely to lead to an underestimate of the impact of malaria interventions.

The last two steps, adjusting for urban*–*rural differences and impact of preventive activities, were not undertaken by Snow et al [6] but were followed by Korenromp [7]. The procedure for estimating the effect of ITNs differs slightly from that used by Korenromp who reduced incidence by 0.5% for each 0.6% increase in ITN use by children <5. However, the correction factors applied are of approximately the same magnitude since rates of ITN use in children <5 are approximately half those of the percentage of households owning at least one ITNs [19]. The adjustments for the impact of preventive activities result in a reduction in the number of cases estimated for the continent of 21%.

***Uncertainty analysis*** An attempt was made to quantify the uncertainty in the malaria incidence rate that could be applied to each category of malaria transmission. A truncated triangular distribution of basic incidence rates was assumed for each age group and category of transmission risk. The median and inter-quartile ranges of the triangular distributions were the same as those of the basic incidence rates shown in Table 1 of the main text. The triangular distributions were truncated so that their lower limit did not fall below 0. In repeated simulations the truncated triangular distribution was found to provide a good approximation for the distribution of basic incidence rates inferred from individual studies. Its use was preferred to deriving a distribution of basic incidence rates from a separate literature review in order to make the results more consistent with those of Snow et al [6].

Palisade @Risk software (version 5.0) was used to sample from the distributions assumed for each parameter. Latin Hypercube sampling was performed 1000 times for each country to yield a distribution for the estimated number of fever and malaria cases for a country, and for the African continent, from which the mean value was taken together with 5% and 95% uncertainty limits for the mean.

***Limitations*** Method 2 gives only a rough guide to the number of malaria cases in any country. Some specific limitations of Method 2 are summarized in Box 2 of the main text.

Table S1. Estimates of populations at risk and intervention (ITN) coverage, used in estimating the number of cases by Method 2.

Table S2. Methods used to estimate malaria incidence for each of 99 countries, with parameter values for Method 1.


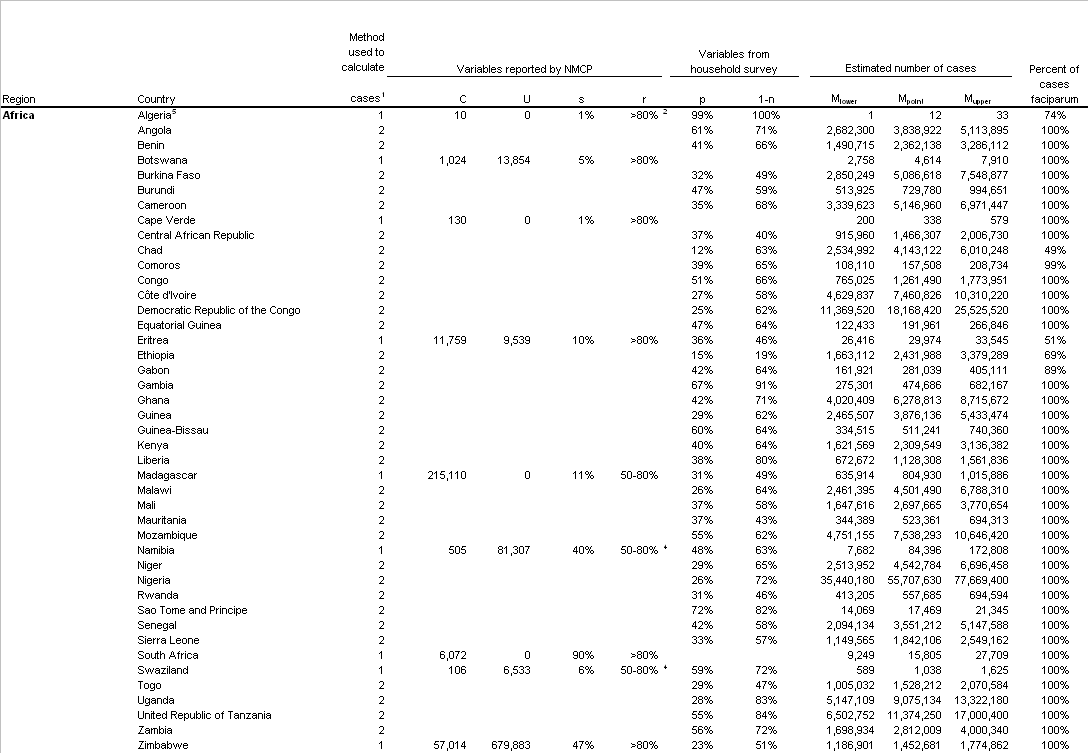


Table S2 (cont.)


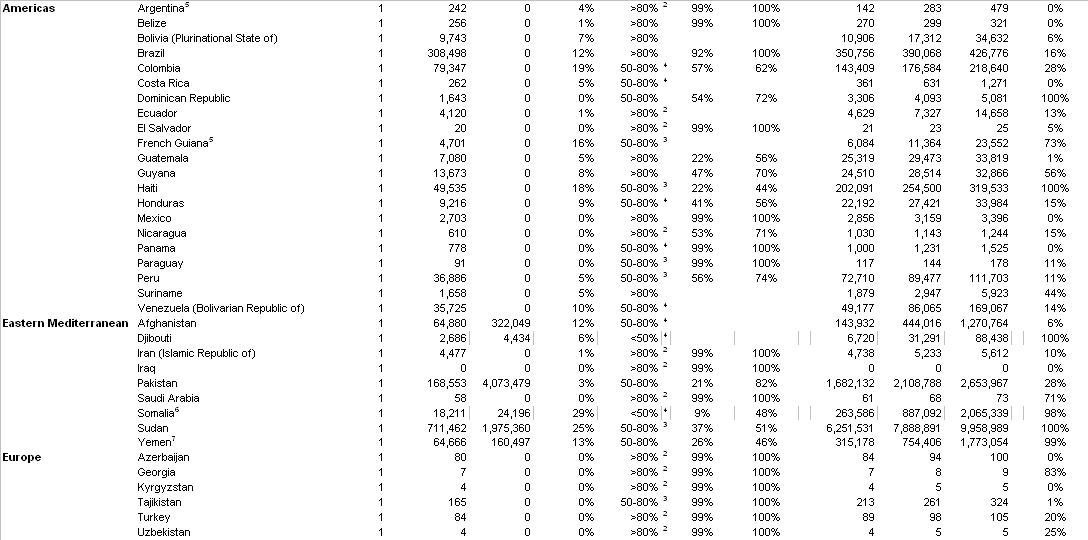


Table S2 (cont.)


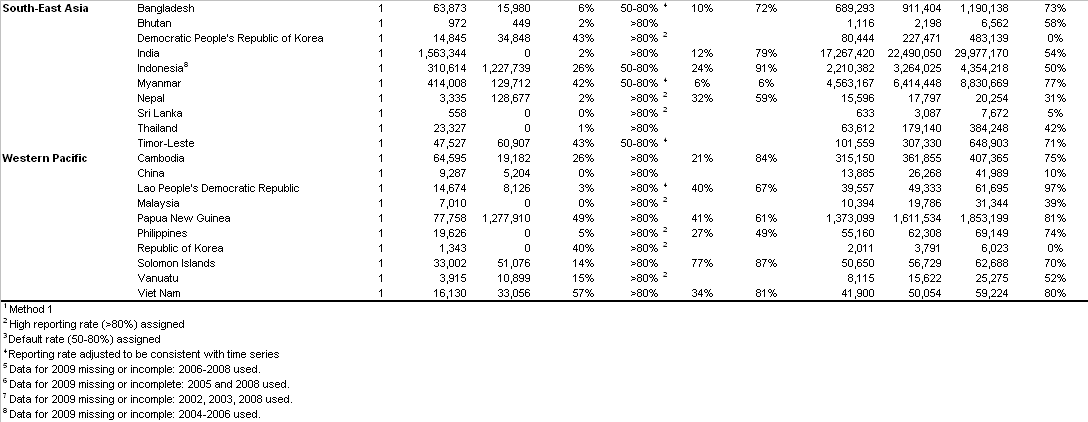


**References**

1. Abba K, Deeks JJ, Olliaro P, Naing CM, Jackson SM, et al. (2011) Rapid diagnostic tests for diagnosing uncomplicated P. falciparum malaria in endemic countries. Cochrane Database Systematic Reviews: CD008122.

2. National Sample Survey Office (NSSO). India.

3. The National Socioeconomic Survey (SUSENAS). Jakarta, Indonesia.

4. Demographic and Health Surveys (DHS). Calverton, Maryland.

5. UNICEF Multiple Indicator Cluster Survey (MICS). UNICEF.

6. Snow RW, Craig MH, Newton CRJC, Steketee RW (2003) The public health burden of *Plasmodium falciparum* malaria in Africa: deriving the numbers. Working Paper 11, Disease Control Priorities Project. Bethesda, Maryland: Fogarty International Centre, National Institutes of Health.

7. Korenromp EL (2005) Malaria incidence estimates at country level for the year 2004 - proposed estimates and draft report Geneva: World Health Organization.

8. Craig MH, Snow RW, le Sueur D (1999) A climate-based distribution model of malaria transmission in sub-Saharan Africa. Parasitology Today 15: 105-111.

9. Omumbo JA, Hay SI, Guerra CA, Snow RW (2004) The relationship between the *Plasmodium falciparum* parasite ratio in childhood and climate estimates of malaria transmission in Kenya. Malaria Journal 3: 17.

10. MARA (2004) Mapping Malaria Risk in Africa.

11. United Nations Population Division (2006) World population prospects: The 2006 Revision. New York: United Nations.

12. United Nations Population Division (2004) World urbanization prospects: The 2003 Revision. New York: United Nations.

13. Carneiro I, Roca-Feltrer A, Schellenberg J (2005) Estimates of the burden of malaria morbidity in Africa in children under the age of five years. Child Health Epidemiology Reference Working Paper. London: London School of Hygiene and Tropical Medicine.

14. Eisele TP, Larsen D, Steketee RW (2010) Protective efficacy of interventions for preventing malaria mortality in children in Plasmodium falciparum endemic areas. International Journal of Epidemiology 39: Suppl 1: 88-101.

15. Lengeler C (2004) Insecticide-treated bed nets and curtains for preventing malaria. Cochrane Database Systematic Reviews: CD000363.

16. Eisele TP, Larsen D, Steketee RW (2010) Protective efficacy of interventions for preventing malaria mortality in children in Plasmodium falciparum endemic areas. International Journal of Epidemiology 39 Suppl 1: i88-101.

17. Flaxman AD, Fullman N, Otten MWJ, Menon M, Cibulskis RE, et al. (2010) Rapid scaling up of insecticide-treated bed net coverage in Africa and its relationship with development assistance for health: a systematic synthesis of supply, distribution, and household survey data. PLoS Medicine 7: e1000328.

18. World Health Organization (2010) World Malaria Report. Geneva: World Health Organization.

19. Korenromp EL, Miller J, Cibulskis RE, Kabir Cham M, Alnwick D, et al. (2003) Monitoring mosquito net coverage for malaria control in Africa: possession vs. use by children under 5 years. Tropical Medicine and International Health 8: 693-703.
